# The Role of Autophagy in Manganese-Induced Neurotoxicity

**DOI:** 10.3389/fnins.2020.574750

**Published:** 2020-09-15

**Authors:** Dong-Ying Yan, Bin Xu

**Affiliations:** ^1^Department of Occupational and Environmental Health, School of Public Health, Jinzhou Medical University, Jinzhou, China; ^2^Department of Environmental Health, School of Public Health, China Medical University, Shenyang, China

**Keywords:** manganese, neurotoxicity, autophagy, alpha-synuclein, endoplasmic reticulum stress, protein S-nitrosylation

## Abstract

Manganese (Mn), an essential micronutrient, acts as a cofactor for multiple enzymes. Epidemiological investigations have shown that an excessive level of Mn is an important environmental factor involved in neurotoxicity. Frequent pollution of air and water by Mn is a serious threat to the health of the population. Overexposure to Mn is particularly detrimental to the central nervous system, leading to symptoms similar to several neurological disorders. Many different mechanisms have been implicated in Mn-induced neurotoxicity, including oxidative/nitrosative stress, toxic protein aggregation, endoplasmic reticulum (ER) stress, mitochondrial dysfunction, dysregulation of autophagy, and the apoptotic cascade, which together promote the progressive neurodegeneration of nerve cells. As a compensatory regulatory mechanism, autophagy plays dual roles in various biological activities under pathological stress conditions. Dysregulation of autophagy is involved in the development of neurodegenerative disorders, with recent emerging evidence indicating a strong, complex relationship between autophagy and Mn-induced neurotoxicity. This review discusses the connection between autophagy and Mn-induced neurotoxicity, especially alpha-synuclein oligomerization, ER stress, and aberrated protein S-nitrosylation, which will provide new insights to profoundly explore the precise mechanisms of Mn-induced neurotoxicity.

## Manganese and Neurotoxicity

Manganese (Mn) is the fifth most abundant metal element overall on earth. It widely exists in ores, oxides, carbonates, and silicates (Horning et al., 2015). Compounds containing Mn have been used in a wide range of industrial processes and commercial products for centuries, such as in antiknock gasoline additives, fungicide additives, steel and stainless-steel production, and aluminum alloy formation (Chen et al., 2016; Parmalee and Aschner, 2016). As an essential micronutrient, Mn is required to synthesize multiple enzymes, including Mn superoxide dismutase enzymes, arginase, glutamine synthetase, phosphoenolpyruvate decarboxylase, and pyruvate carboxylase. A proper level of Mn is crucial for human health, but excessive exposure to Mn may result in a neurotoxic disorder known as manganism (Horning et al., 2015; Parmalee and Aschner, 2016), which has symptoms resembling those of Parkinson’s disease (PD), involving the expression of cognitive, motor, and emotional deficits. Population studies have shown that long-term inhalation of Mn-containing gases at a level >5 mg/m^3^ can increase the occurrence of neurotoxicity (Khan et al., 2012; O’Neal and Zheng, 2015). The World Health Organization (WHO) reports that drinking water containing Mn at a level >400 μg/L can increase the risk of neurological disorders (Khan et al., 2012). Epidemiological survey results indicate that chronic Mn exposure in the occupational environment is the main pathogenic cause of clinical diagnoses of manganism in patients (O’Neal and Zheng, 2015).

Mn can pass through the blood–brain barrier (BBB) and mainly accumulate in the striatum, globus pallidus (GP), and the substantia nigra (SN), with either Mn overexposure or deficiency capable of causing neurological dysfunction (Parmalee and Aschner, 2016; Balachandran et al., 2020). Investigations of Mn overexposure over the past decade have mainly focused on oxidative/nitrosative stress, endoplasmic reticulum (ER) stress, energy failure, nerve cell apoptosis, mitochondrial dysregulation, alpha-synuclein (α-syn) oligomerization, alteration of neurotransmitter metabolism, and calcium dyshomeostasis (O’Neal and Zheng, 2015), while Mn deficiency is mostly due to the reduced activity of multiple Mn-dependent enzymes. Emerging evidence indicates that oxidative stress-mediated apoptotic cell death and epigenetic mechanisms have been implicated in Mn-induced neurotoxicity: (1) Mn accumulates in mitochondria causing inhibition of complex I/II and F1ATPase and disrupts Ca^2+^ homeostasis-activated ATP production, inducing apoptosis due to loss of mitochondrial potential with the release of cytochrome C and caspase-3 activation; (2) caspase-3 proteolytically activates PKCδ that can activate the ERK/MAPK pathway, which enhances the transcription of PKCδ-dependent pro-apoptotic genes resulting in mitochondrial-mediated apoptotic cell death; (3) Mn may also cause the ER stress that activates caspase-12 which culminates in apoptotic cell death; (4) the generation of ROS/RNS by Mn causes damage to DNA/RNA/protein/lipid and release inflammatory mediators that induce apoptosis; (5) cytosolic α-syn interacts with antiapoptotic 14-3-3 protein, making pro-apoptotic BAD cause mitochondrial impairment (Tarale et al., 2016). Mn exhibits a propensity to damage dopaminergic neurons. Autophagy affords neuroprotection via eliminating defective cell structures and the degradation of the disease-related mutant protein. Increasing evidence also suggests that there is a crosstalk between autophagy and apoptosis. Dysregulation of autophagy has been shown to contribute to Mn-induced apoptosis (Zhou et al., 2018; Zhang et al., 2019), but the molecular mechanisms underlying this neurotoxicity have yet to be fully elucidated.

## Overview of Autophagy

**FIGURE 1:**
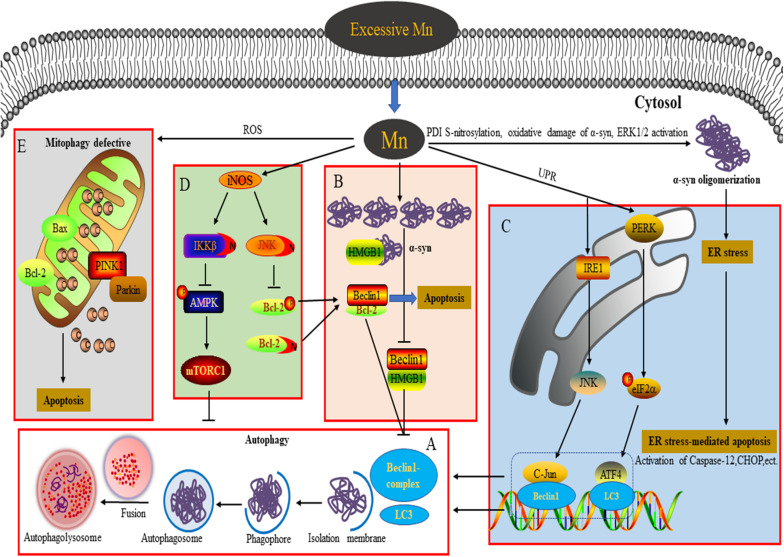
Connection between autophagy and neurotoxicity induced by an excessive level of Mn. **(A)** Process of autophagy involved in the degradation of α-syn overexpression. **(B)** Overexpression of α-syn induced by Mn inhibits autophagy by disrupting Beclin1-HMGB1 binding. **(C)** Autophagy plays a protective role against ER stress-mediated apoptosis by activating the PERK and IRE1 pathways during Mn-induced neurotoxicity. **(D)** Aberrant protein S-nitrosylation (of JNK, Bcl2, and IKKβ) is involved in dysfunctional autophagy leading to Mn-induced nerve cell damage. **(E)** An excessive level of Mn triggers defective mitophagy, inducing mitochondrial-mediated apoptosis.

Autophagy is an evolutionarily conserved process in eukaryotes that degrades damaged organelles and macromolecules and maintains cellular homeostasis in response to conditions of stress (Ghavami et al., 2014; Yu et al., 2018). There are three forms of autophagy: macroautophagy, microautophagy, and chaperone-mediated autophagy. Generally, what is termed “autophagy” refers to macroautophagy (Yu et al., 2018). The process of autophagy proceeds through four steps (Figure 1A): (1) separation membrane formation; (2) autophagosome formation; (3) autophagosome transport and fusion with the lysosome; and (4) degradation of components in the autophagolysosome (Yu et al., 2018). A large number of autophagy-related proteins (ATGs) coded by *ATG* genes are involved in the autophagy-related degradation process (Feng et al., 2014), including polyglutamine-induced microtubule-associated protein 1 (MAP1) light chain 3 (LC3, the mammalian homolog of ATG8), a marker of autophagy, which lipidates the unbound LC3-I to the membrane-bound LC3-II and is necessary for synthetic processes during autophagy; Beclin1/ATG6, which is involved in autophagy initiation and nucleation; and p62, which is involved in the recognition process that links autophagosomes and cargo during the scaffolding and formation of the autophagosome. Autophagy is a dynamic and complex biological process from autophagosome synthesis to degradation, which is in a continual state of flux. Basal levels of autophagy run are low, but autophagy induction processes can be quickly activated to promote cellular survival by maintaining adequate amino acid pools, and cellular energy levels during extracellular insults, such as starvation (Yu et al., 2018). Accumulating evidence indicates that autophagy functions as a double-edged sword, with appropriate activation of the autophagic pathway playing a cytoprotective role under pathological conditions, but overactivation or suppression of autophagy resulting in aggravation of pathological lesions via the induction of autophagy-dependent programmed cell death (Ghavami et al., 2014; Parzych and Klionsky, 2014). An increasing number of studies show that the dysregulation of autophagy is closely linked with the occurrence and progression of neurodegenerative diseases (Ghavami et al., 2014; Lapierre et al., 2015). Hence, an in-depth understanding of the role of autophagy can provide novel perspectives on the study of Mn-induced neurotoxicity.

Numerous studies have demonstrated that Mn exposure increases expression of Beclin1, LC3-II, and p62, leading to the activation of autophagy. However, the upregulation of these proteins by Mn can be the result of either increased autophagic flux or blocked autophagic clearance. An integrated approach to investigating autophagy would require a series of dynamic monitoring methodologies (most likely transfection of a plasmid encoding a tandem GFP-mCherry-LC3B fusion protein to evaluate autophagy flux) with pharmacological manipulation using positive modulators, such as melatonin or rapamycin (Rap), and negative modulators of autophagy, such as LY294002, chloroquine (CQ), bafilomycin A (BafA), or 3-methyladenine (3-MA) (Bryan et al., 2019). In their research into Mn-induced neurotoxicity, Zhang and colleagues found that at an early stage of autophagy (4–12 h after injection of 1 mol/L MnCl_2_ into the right striatum) in Mn-exposed rats, the adaptive activation of autophagy reflects transient protection against Mn-induced neurotoxicity. However, at a later stage (1–28 days post-injection), suppression of autophagy is associated with Mn-induced dopaminergic neuron neurodegeneration (Zhang et al., 2013). These findings indicate that the varying duration of exposure to Mn should be taken into account in understanding the process of Mn-induced autophagy. In a Huntington’s disease (HD) cell model, intracellular dyshomeostasis of Mn, which is required as an essential trace element, is associated with defective autophagy. The increase in the level of p62 caused by Mn can be abrogated by PI3K inhibition (with LY294002) but is not changed by mTOR inhibition (with Rap), indicating that the Mn-induced increase in p62 is PI3K-dependent. Treatment with lysosomal autophagy inhibitors (namely, CQ and BafA) can cause the accumulation of LC3-II puncta and increase the net uptake of Mn, effectively attenuating defects in Mn-induced autophagy in ST*Hdh*^Q111^ cells (Bryan et al., 2019). Melatonin/Rap enhances autophagy to retard Mn cytotoxicity by ameliorating lysosomal dysfunction in BV-2 cells (Porte Alcon et al., 2020). More studies are required in the future to explore the mechanisms underlying the impingement of Mn on autophagy in different levels of intracellular Mn at which Mn-induced neurotoxicity leads to distinct detrimental outcomes.

## Autophagy and Mn-Induced α-Syn Oligomerization

Previous studies into Mn-induced neurotoxicity have indicated that an increase of α-syn oligomers plays a key role in Mn-induced neurocyte injury (Xu et al., 2014a,b; Peres et al., 2016; Yan et al., 2019a). Unlike the pathogenic α-syn involved in PD, the α-syn induced by Mn takes the form of soluble and stable intermediary oligomers, rather than insoluble and mature α-syn fibrils (Peres et al., 2016). As a small protein, α-syn plays an important role in mediating various physiological functions, such as synaptic plasticity, regulation of vesicle transport, dopaminergic neurotransmission, and anti-oxidative stress and anti-apoptotic functions (Peres et al., 2016). Besides, α-syn is considered to act as a Mn store, protecting cells against Mn-induced neurotoxicity in the early phase by attenuating the apoptotic cascade activation, interfering with PKCδ, and reducing p300 histone acetyltransferase activity (Harischandra et al., 2015). In contrast, overexpression, or oligomerization of α-syn promotes apoptotic cell death by perturbing membrane integrity, activating NF-κB, p38MAPK, and caspase signaling (Peres et al., 2016). Additionally, α-syn directly binds to the kinase domain of the neurotrophic receptor TrkB and disturbs the mediation of signal transduction by brain-derived neurotrophic factor, resulting in learning and memory impairment (Kang et al., 2017; Vijayan et al., 2019). Oxidative damage of α-syn protein, S-nitrosylation of protein disulfide isomerase (PDI), and extracellular signal-regulated kinase (ERK1/2) activation have been suggested to be dose-dependently involved in Mn-induced α-syn overexpression and oligomerization (Cai et al., 2010; Xu et al., 2013, 2014a). Autophagy is a critical degradation pathway that is important for the control of intracellular proteostasis, with current evidence from multiple studies indicating the complex relationship between autophagy and α-syn. For example, Winslow et al. (2010) found that α-syn can interact with Rab1a to suppress macroautophagy, while in α-syn-overexpressing cells, Tanik et al. (2013) observed an accumulation of enlarged autophagosomes and lysosomes. Dopaminergic neurons are particularly susceptible to changes in the levels of α-syn, which may be a significant reason for autophagy dysfunction (Zhang et al., 2013). Our team has found that exposure of α-syn gene knockout mice to Mn results in excessive autophagy and aggravation of apoptosis, highlighting to some degree the physiological role of α-syn in reducing excessive autophagy. Moreover, when we applied Rap and 3-MA *in vivo*, we found that inhibition of autophagy by 3-MA increases apoptosis and the level of α-syn oligomers. However, Rap exerts opposing effects, further emphasizing the major role of α-syn oligomerization in Mn-induced neurotoxicity (Yan et al., 2019a). The potential molecular mechanism underlying this may be associated with the failure of Beclin1-dependent induction of autophagy. In SH-SY5Y cells, we found that the overexpressed α-syn induced by Mn preferentially binds to high mobility group protein B1 (HMGB1), disrupting Beclin1–HMGB1 autophagic induction, and exacerbating apoptosis via an increase of Beclin1–Bcl2 binding (Yan et al., 2019b). These results partly reveal the potential mechanism by which overexpressed α-syn affects Mn-induced autophagy dysfunction (Figure 1B). These events cannot rule out a partial effect of Mn itself. In addition, the state (subcellular localization and redox form) of HMGB1 affects autophagy induction. It is controlled by various factors, including positive regulators (autophagy activators, such as ULK1, MAPK, and NACC1) and negative regulators (autophagy inhibitors, such as Tp53, α-syn, IFI30, MIR34A, and MIR22) (Sun and Tang, 2014). HMGB1 is able to regulate Bcl2 phosphorylation via ERK/MAPK signaling, inducing the activation of autophagy (Tang et al., 2010). Furthermore, Mn-induced changes in α-syn localization (for example, in the intracellular compartment vs. extracellular exosomes) likely influence other aspects of α-syn biology, for example, the microglial-mediated neuroinflammatory response (Harischandra et al., 2018, 2019). ATP13A2, a P-type ATPase located in lysosomes, has also been implicated in the regulation of intracellular Mn homeostasis and the toxicity of a-syn aggregates (Fleming et al., 2018; Ugolino et al., 2019). In order to extend our understanding further, the exact regulatory mechanisms underlying Mn-induced autophagy dysfunction and α-syn oligomerization deserve intensive exploration in future.

## Autophagy and Mn-Induced ER Stress

ER is the primary cellular location responsible for the synthesis, folding, assembly, post-translational modification, and transportation of nascent proteins. Under conditions of protein unfolding or an overload of misfolded proteins in the ER (triggering ER stress), the unfolded protein response can be subsequently activated to protect against the toxic protein via the ER-associated ubiquitin/proteasome degradation (ERAD) pathway (Velentzas et al., 2013). However, persistent ER stress can trigger ER-stress-mediated cell death through various mechanisms, including activation of caspase-4 and caspase-12 (specifically localized in the ER), and upregulation of the expression levels of various proteins, such as pro-apoptotic C/EBP homologous protein (CHOP), growth arrest and DNA damage 34 (GADD34), and poly-ADP-ribose polymerase (PARP) (Song et al., 2017). ER stress receptors are composed of PKR-like ER kinase (PERK), activating transcription factor 6 (ATF6), and inositol-requiring enzyme 1 (IRE1), which regulate the dual protective and compensatory role of the unfolded protein response in ER stress (Roussel et al., 2013). Converging evidence indicates a close relationship between autophagy activation and ER stress (Kouroku et al., 2007; Song et al., 2017; Liu et al., 2018, 2020a,b). In the course of the progressive accumulation of unfolding/misfold proteins, PERK dissociates from the molecular chaperone 78 glucose-regulated protein (GRP78), leading to activation of PERK in the ER (Roussel et al., 2013). Activation of PERK represses the synthesis of unfolded/misfolded proteins and phosphorylates eukaryotic translation initiation factor 2 α (elF2α) (Velentzas et al., 2013). Momoi et al. reported that PERK/elF2α phosphorylation is involved in the conversion of LC3, from LC3-I to LC3-II form (Kouroku et al., 2007). A previous study from our lab showed that Mn induces ER stress via activation of PERK/eIF2α/ATF4 and IRE/X-box binding protein 1 (Xbp1) signaling pathways, as well as apoptosis, accompanied by a dose-dependent increase of GRP78/94, CHOP, and caspase-12 in cultured organotypic brain slices (Xu et al., 2014b). As a regulatory response to ER stress, autophagy activation can be regulated by multiple signaling pathways to protect against ER stress-mediated apoptosis (Song et al., 2017). The interaction between GADD34 (which can be upregulated by CHOP and ATF4) and protein phosphatase (PP1) can lead to dephosphorylation of eIF2α to promote excessive ER stress-mediated apoptosis (Roussel et al., 2013). In addition, ATF4, a downstream transcription factor in the PERK signaling pathway, is able to regulate the expression of LC3 (Kouroku et al., 2007). In SH-SY5Y cells, Mn treatment (100 μM) enhances the binding of ATF4 to the LC3 promoter and increases LC3 mRNA expression to mitigate apoptosis via activation of the PERK signaling pathway, indicating that the activation of autophagy plays a protective role in response to Mn-induced ER stress-mediated apoptosis (Figure 1C; Liu et al., 2020b). As a specific substrate of IRE, the transformation of Xbp1 into sXbp1 via splicing is the result of IRE1 signaling activation, after which sXbp1 participates in the regulation of ERAD-related protein transcription (Roussel et al., 2013). Moreover, the key downstream molecules of IRE1 signaling, including apoptosis signal-regulating kinase1 (ASK-1) and Jun N-terminal kinase (JNK), have been implicated in ER stress-induced apoptosis and autophagy (Song et al., 2017). In SH-SY5Y cells pretreated with SP600125 (a JNK specific inhibitor), the activation of autophagy by Mn (100 μM) appears to be inhibited and neurocyte injury is subsequently aggravated; this can be partly attributed to a weakening of the interaction between c-Jun and the Beclin1 promoter, thereby leading to a failure of a protective autophagic response against apoptosis (Figure 1C; Liu et al., 2020a). The activation of JNK signaling is mediated by an interaction between ASK-1 and tumor necrosis factor (TNF) receptor-associated factor 2 (TRAF2), with the expression of both ASK-1 and TRAF2 regulated by IRE1 (Liu et al., 2020a). These findings reveal the intrinsic relationship between autophagy and ER stress-related signaling in Mn-induced neurocyte injury.

## Autophagy and Mn-Induced Nitrosative Stress

Numerous studies have demonstrated that nitrosative stress participates in the etiology of diverse neurodegenerative diseases such as PD, Alzheimer’s disease, and manganism (Nakamura et al., 2015). Both neuronal NOS (nNOS) and inducible NOS (iNOS) are connected with aberrant NO overproduction in neurodegenerative disorders. As an essential cofactor for many enzymes (such as arginase and agmatinase), a deficiency of bioavailable Mn and the resulting reduced arginase activity are involved in striatal neurodegeneration in HD striatal mouse and cell models. Overexposure to Mn has been shown to increase iNOS, leading to increases in NO and glial activation via arginase 2 (ARG2), the arginase enzyme (Bichell et al., 2017). These findings show an intricate connection between Mn bioavailability, Mn-dependent enzyme activity (namely of arginase), and NO production. Under pathological conditions, an increased NO level triggers nitrosative stress and aberrant protein S-nitrosylation, resulting in various detrimental effects, including protein misfolding, mitochondrial fragmentation, synaptic dysfunction, apoptosis, or autophagy. Two key pathways have been implicated in the role of NO bioactivity in autophagy: JNK1/Bcl-2/Beclin1 and IKKβ/AMPK/mTORC1. Under conditions of stress, JNK1 is activated to phosphorylate Bcl2 and cause it to dissociate from Beclin1, inducing autophagy (Haldar and Stamler, 2011; Zhou et al., 2015). However, excessive NO has been found to inhibit autophagy in association with S-nitrosylation of JNK1 (at C116), limiting its activity. Additionally, S-nitrosylation of Bcl2 has been implicated in the coordinated inhibition of autophagy by limiting the dissociation of Beclin1 via phospho-Bcl2 reduction (Haldar and Stamler, 2011). Previous studies from our lab have revealed that aberrant increases in the S-nitrosylation of JNK1 and Bcl-2 are further involved in the dysregulation of Mn-induced autophagy. In addition, the NO-mediated S-nitrosylation of Bcl-2 by Mn directly enhances the interaction between Beclin1 and Bcl2 in SH-SY5Y cells, leading to the inhibition of autophagy (Ma et al., 2017). Furthermore, the inhibition of NO-mediated autophagy is also associated with S-nitrosylation of IKKβ (Nakamura et al., 2013; Zhou et al., 2015), while activation of IKKβ can induce phosphorylation of AMPK, leading to the inactivation of mTORC1 (a classical inhibitor of autophagy) and a subsequent activation of autophagy. However, the NO-mediated S-nitrosylation of IKKβ inhibits its kinase activity, meaning that it is unable to inactivate mTORC1 and thereby blocking autophagic flux (Haldar and Stamler, 2011). *In vivo* results from C57/BL6 mice and *in vitro* SH-SY5Y cell studies have shown convincingly that in addition to S-nitrosylation of JNK and Bcl-2, exposure to Mn (300 μmol/kg or 200 μM) also significantly increases the S-nitrosylation of IKKβ, thereby leading to autophagy inhibition via the IKKβ/AMPK/mTORC1 signaling pathway (Figure 1D; Ma et al., 2020). Thus, following S-nitrosylation, the crucial signaling molecules JNK, Bcl-2, and IKKβ are considered to be causal factors in Mn-induced nitrosative stress and autophagy dysfunction.

Protein S-nitrosylation can be reversed by denitrosylation enzymes, such as PDI, S-nitrosoglutathione reductase, and thioredoxin, which remove NO from S-nitrosylated cysteine residues, ameliorating nitrosative stress under pathological conditions (Nakamura et al., 2013, 2015). In Mn-treated brain slices, dose-dependent increases of S-nitrosylated PDI and α-syn oligomerization have been shown to be involved in Mn-induced nerve cell injury (Xu et al., 2014a). Excessive NO can also trigger mitochondrial dysfunction, contributing to the occurrence of neurodegenerative diseases. Normal mitochondria play a crucial role in the maintenance of redox balance and neuronal synaptic activity (Nakamura et al., 2013). Mn overexposure can cause mitochondrial dysfunction accompanied by the release of cytochrome C and caspase 3/7 activation, resulting in the death of dopaminergic neurons. Notably, Mn-induced mitochondrial dysfunction may occur after acute cytotoxic thresholds have been reached (Warren et al., 2020). Additionally, S-nitrosylation of dynamin-related protein 1 (Drp1) has been found to result in excessive mitochondrial fission and the aggravation of Aβ-induced synaptic damage (Nakamura et al., 2013). Parkin, an E3 ubiquitin ligase, plays a neuroprotective role in neurodegenerative diseases through various processes, such as the degradation of neurotoxic proteins via the ubiquitin-proteasome system (UPS) and ERAD, and suppression of P53 transcription (Nakamura et al., 2013). Recently, Parkin has been reported to be involved in mitophagy, with emerging evidence indicating that PTEN induced putative kinase 1 (PINK1) recruits Parkin to the damaged mitochondrial membrane and then ubiquitinates the mitochondrial outer membrane protein, promoting the removal of damaged mitochondria by mitophagy (Zhang et al., 2016). However, under pathological stress, the mitochondrial quality control machinery can be compromised because of excessive production of NO, which is associated with a lack of Parkin E3 ligase activity and leads to S-nitrosylation of Parkin (Nakamura et al., 2013). These findings support the conclusion that aberrant S-nitrosylation contributes to autophagy dysfunction and neurotoxicity. Under conditions of Mn-induced mitochondrial dysfunction, levels of PINK1 and Parkin are significantly increased in a dose-dependent manner, accompanied by defective mitophagy (Figure 1E; Liu et al., 2019). Trehalose pretreatment has been shown to attenuate Mn-induced mitochondrial dysfunction by providing resistance to oxidation and improving autophagic flux (Liu et al., 2019). However, the effects of protein S-nitrosylation (of Parkin and Drp1) on Mn-induced mitochondrial dysfunction are far more complex than this, and the potential mechanisms underlying the effects of NO remain to be elucidated.

## Conclusion

Direct evidence for a causal link between autophagy and Mn neurotoxicity is still limited, despite the strong connection in the aspects of nerve cell apoptosis, α-syn oligomerization, ER, and nitrosative stresses. Either deficiency Mn or excessive Mn influx can cause autophagy dysfunction contributing to neurodegeneration, which may provide new insights to comprehensively study autophagy dysfunction mechanism in terms of Mn uptake, Mn-dependent enzyme activity, α-syn biology function, and lysosome function. Furthermore, autophagy is regulated by multiple signal kinases and various transcription factors and shares molecules with apoptosis signaling, which may carefully affect cell fate in response to Mn. However, the exact molecular mechanism is not fully clarified. Besides this, autophagy-related protein post-transcriptional modification will also be another essential field for studying the role of autophagy in Mn neurotoxicity. Hopefully, regulation of the neuroprotective role of autophagy may act as a potent impetus for therapy Mn-associated neurodegeneration in the future.

## Author Contributions

D-YY and BX conceptualized the theme of the review and wrote the manuscript together. Both authors contributed to the article and approved the submitted version.

## Conflict of Interest

The authors declare that the research was conducted in the absence of any commercial or financial relationships that could be construed as a potential conflict of interest.
